# Oatmeal

**Published:** 1875-06

**Authors:** 


### OATMEAL.

Oatmeal is principally used in two ways—for the making of mush or porridge and of oat-cakes. Porridge is a principal article of food of the Scottish peasantry, generally accompanied with milk, when milk can be obtained, although when milk is scarce, butter is sometimes used, sometimes sugar, and sometimes molasses. For most people in a sound condition of health there is no more wholesome article of food than oatmeal mush and milk; none contains a larger proportion of flesh-forming and heat-producing substances; while to almost all who have ever been accustomed to its use it is extremely palatable. Generally speaking, there is no better article of food for the nursery, none more likely to maintain a healthy condition of the stomach or to give vigor to the frame, although there are exceptional cases, both among the young and among adults, in which the use of mush or porridge is unsuitable, producing painful distension of the stomach and indigestion. While the caprices of children ought not to be heeded in such a matter, the actual conditions of their constitution ought to be carefully observed and regarded. Mush is in general made by simply boiling oatmeal in water, stirring all the while to prevent singeing, and to secure the thorough mixture of the oatmeal and water into a homogenous mass without knots. The quality of mush very much depends on the amount of boiling which it receives. It can not be too thoroughly boiled. Imperfectly boiled oatmeal is a very coarse article of food; and, unfortunately, much of the article used by the poorer classes in Scotland and elsewhere is of this character, and that prepared for the nursery is often no better, through the carelessness of servants, who wish to get through their work with as little trouble as possible. It is not nearly so digestible, and therefore not so nutritious, as when really well made. A common mistake in the making of mush must also here be noticed, as tending much to the deterioration of its quality—the adding of meal by degrees, while the boiling goes on, until the proper thickness is acquired, the result being that part of the meal is imperfectly boiled. The cook ought to know the proper proportions of meal and water—knowledge not very difficult to acquire—and mix them at once, so that all the meal may be equally boiled. But it is to be observed that the water must be boiling before the meal is put in, which is not to be introduced in a mass, but, as it were, strained through the fingers handful by handful, as quickly as possible.

---

Children need sunshine quite as much as flowers do. Half an hour daily is not enough. Several hours are required. The most beautiful flowers that ever studded a meadow could not be made half so beautiful without days and days of the glad light that streams through space. Light for children. Sunshine for the little elves that gladden this otherwise gloomy earth. Deal it out in generous fullness to them. Let the nursery be in the sunshine. Better plant roses on the dark side of an iceberg than rear babies and children in rooms and alleys stinted of the light that makes life.

